# Effect of Heat Treatment on Cr_2_Nb Phase and Properties of Spark Plasma Sintered Cu-2Cr-1Nb Alloy

**DOI:** 10.3390/ma13122860

**Published:** 2020-06-25

**Authors:** Xueqian Lv, Zuming Liu, Ting Lei, Quan Li, Yake Ren, Xu Zhou, Zejie Zhang

**Affiliations:** State Key Laboratory of Powder Metallurgy, Central South University, Changsha 410083, China; lvxueqian@163.com (X.L.); qli019@sjtu.edu.cn (Q.L.); renyake@csu.edu.cn (Y.R.); zhou.xu@csu.edu.cn (X.Z.); fyyzzj@csu.edu.cn (Z.Z.)

**Keywords:** Cu-2Cr-1Nb alloy, heat treatment, multi-scale Cr_2_Nb phase, heat treatment hardening, tensile strength, conductivity

## Abstract

Achieving a good match between strength and conductivity is a challenge of the development of the high-performance Cu-Cr-Nb alloy for aerospace and fusion energy. The effect of heat treatment on Cr_2_Nb phase, strength and conductivity of spark plasma sintered (SPSed) Cu-2Cr-1Nb (at%) alloy was investigated. The results illustrated that Cr_2_Nb phase of Cu-2Cr-1Nb alloy can be regulated by heat treatment, multi-scale Cr_2_Nb phase with sizes of 0.10–0.50 μm, 30–100 nm and less than 30 nm was obtained, and the strength and conductivity were significantly increased after heat treatment at 500 °C for 2 h, the room temperature tensile strength and conductivity were 332 MPa and 86.7% IACS, 2.5% and 34.8% higher than those of as-SPSed alloy; the tensile strength at 700 °C was 76 MPa. Increasing heat treatment temperature and time, the tensile strength of the alloy was reduced by 1.5%, 4.3% and 12.3% after heat treatment at 500 °C, 700 °C and 950 °C for 72 h. The good match between strength and conductivity of Cu-Cr-Nb alloy was obtained by reducing the content of alloying elements (Cr and Nb) and microstructure regulation. This approach can be used to prepare structural/functional materials with excellent strength and conductivity.

## 1. Introduction

High-strength and high-conductivity copper alloy is one of the key structural/functional materials used in many high-tech fields, such as aerospace, energy and so on [1,2]. How to improve the strength and conductivity simultaneously and obtain a good match between strength and conductivity are the focus topics in the development of copper alloys with high performance. Many excellent copper-based materials have been developed, such as Cu-Ag-Zr [3,4], Cu-Cr-Zr [5,6,7,8], Cu- Zr [9], Cu-Cr [10,11], Cu-Al_2_O_3_ [12], Cu-Ni-Si-Cr [1], Cu-Cr-Nb [2,13,14,15,16] and so on. However, for high-heat-flux structural and functional materials in aerospace and fusion energy, there are also high requirements for thermal stability, in addition to strength and conductivity; the applications of many high-strength and high-conductivity copper alloys are limited due to their poor microstructural stability and the over-coarsening of second phase at high temperature.

Cu-Cr-Nb alloy exhibits superior comprehensive properties, such as strength, creep, fatigue, conductivity and microstructural stability, and is one of the most excellent high-heat-flux structural materials for aerospace and fusion energy [1,17,18,19,20,21,22,23]. Microstructure, especially the amount and size of second phases, plays a vital role in the properties of Cu-Cr-Nb alloys [24,25,26,27,28]. Reasonably designing the composition of the alloy and effectively regulating the size of Cr_2_Nb phase are the key to the development of high-performance Cu-Cr-Nb alloy. Dhokey et al. [29,30] reported that a Cu-8Cr-4Nb (at%) alloy prepared by casting, in which the Cr_2_Nb phase was with a size of 0.7–7 μm. Guo et al. [15] regulated microstructure of casted Cu-0.47Cr-0.16Nb (wt%) alloy by homogenization heat treatment, cold rolling (CR) and aging treatment, and obtained Cr_2_Nb phase with the average size of 0.7 μm around. Yang et al. [2] prepared a Cu-2Cr-1.35Nb-0.15Zr (wt%) by drop-casting, deformation processing and heat treatment. The size of the Cr_2_Nb phase in this alloy was 0.3–1 μm, and the conductivity of the alloy was less than 60% IACS. In order to regulate Cr_2_Nb phase and improve the properties of the alloy, Ellis et al. [14] prepared a Cu-Cr-Nb alloy strip by chill block melt spinning (CBMS). The Cr_2_Nb phase in the alloy strip was fine and distributed in the Cu matrix uniformly, and the alloy strip had a combination of high strength, high conductivity and good microstructural stability. Anderson and Ellis et al. fabricated Cu-8Cr-4Nb, Cu-4Cr-2Nb and Cu-8Cr-4Nb (Zr) alloys by hot extrusion (HE) [13,31], hot isostatic pressing (HIP) [21], vacuum hot pressing (VHP) [16,32,33], spark plasma sintering (SPS) [34] and plasma spraying [35] using atomized alloy powders. The average size of Cr_2_Nb phase in Cu-8Cr-4Nb (at%) and Cu-4Cr-2Nb (at%) alloys was about 0.93 μm and 0.78 μm, respectively, and the conductivity of the alloys was both less than 75% IACS. Anderson [13,36] and Shukla [37] investigated the effect of heat treatment on microstructure and properties of Cu-8Cr-4Nb alloy. The fine Cr_2_Nb phase was formed by heat treatment, and the conductivity of the alloy was less than 76% IACS. To further improve the properties of Cu-Cr-Nb alloy, Anderson [13,27,38] and Shukla [34,39] regulated microstructure of the atomized Cu-8Cr-4Nb and Cu-4Cr-2Nb alloy powder by mechanical alloying. The size of Cr_2_Nb phase in the alloys prepared from mechanically alloyed powder decreased significantly, and the mechanical properties of the alloy were improved obviously, but the conductivity was reduced. Therefore, how to effectively regulate microstructure of Cu-Cr-Nb alloy, especially the size of Cr_2_Nb phase, and realize the good match of strength and conductivity, is still the key problem to be solved in the development of high-performance Cu-Cr-Nb alloys.

In our previous work, a Cu-2Cr-1Nb (at%) alloy was fabricated by SPS with close-coupled argon gas atomized alloy powder [40,41], the size of Cr_2_Nb phase decreased obviously, but the strength and conductivity still had room for improvement. This work aimed at improving the properties of the SPSed Cu-2Cr-1Nb alloy by microstructure regulation. The strength and conductivity were simultaneous improved through regulating multi-scale Cr_2_Nb phase of the SPSed Cu-2Cr-1Nb alloy by heat treatment, based on regulating microstructure of Cu-Cr-Nb alloy by reducing content of alloying elements Cr and Nb, rapid solidification by close-coupled argon gas atomization [40] and rapid densification by SPS [41]. This approach has a great significance for the development of high-performance Cu-Cr-Nb alloys.

## 2. Experimental

The Cu-2Cr-1Nb alloy was fabricated by a SPS system (HPD25-3, FCT, Rauenstein, Germany) using close-coupled argon atomized powder [40,41]; the composition of powder is shown in Table 1, and the particle size of powder was below 100 mesh (150 μm). The sintering temperature, sintering pressure, holding time and heating rate were 950 °C, 50 MPa, 15 min, and 100 °C·min^−1^, respectively, and the vacuum degree during SPS was higher than 1 Pa. The prepared alloy was a cylinder with a diameter of 40 mm and height of about 10 mm. The alloys were heat-treated at 500 °C, 700 °C, and 950 °C in a vacuum environment (vacuum higher than 10^−2^ Pa), using a resistance furnace (KSL-1400×, Kejing, Hefei, China), and the heat treatment times were 1 h, 2 h, 3 h, 5 h, 24 h, and 72 h, and the cooling method was water cooling.

The microstructure of the alloy was observed by a scanning electron microscopy (SEM) (Quanta FEG 250, FEI, Hillsboro, OR, USA) and a transmission electron microscopy (TEM) (JEM-2100F, JEOL, Tokyo, Japan). The size of second phase was analyzed by Nano Measurer software (V1.2, Fudan University, Shanghai, China). The SEM samples were prepared by hot-mounting and standard metallographic technique, and were etched by FeCl_3_+HCl reagent (>99%, Tianjin Guangfu Reagent Co., Ltd, Tianjin, China). The TEM samples were prepared by mechanically grounding foils to 60 µm, punching subsequently into discs with diameter of 3 mm, and then twin-jet electro-polished by a electrolytic double spray thinner (Tenupol-5, STRURES, Hovedstaden, Denmark) with an operating voltage of 20.5 V. The etching solution was 24 vol% nitric acid/methanol solution.

In terms of ISO 6892-1: 2016 [42] and ISO 6892-2: 2018 [43], the tensile properties of the alloy were tested by an electronic universal testing machine (Instron-3369, Illinois Tool Works Inc., Norwood, MA, USA) in the air; the test temperature was 25 °C and 700 °C; the strain rate was 1.0 mm·min^−1^. The average of three measured values was taken as the measured tensile strength of the alloy. The electrical conductivity of the alloy was measured by a digital conductivity meter (D60 K, Xiamen Xinbote Technology Co., Ltd., Xiamen, China). Each sample was measured at three points, and the average value was selected as the measured value of conductivity.

## 3. Results and Discussion

### 3.1. The Microstructure of the As-SPSed Cu-2Cr-1Nb Alloy

**Figure 1:**
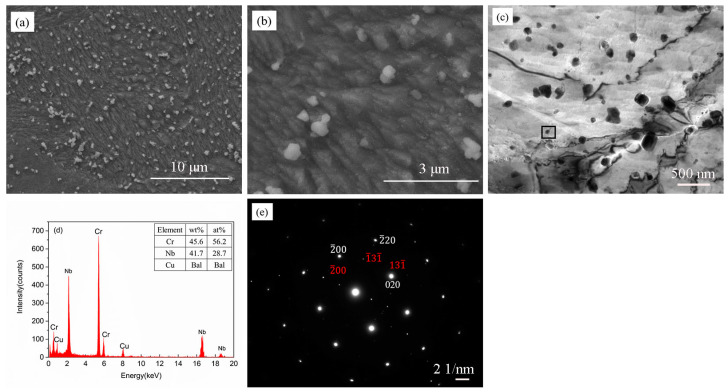
Microstructure of as-SPSed Cu-2Cr-1Nb alloy; (**a**,**b**) SEM micrograph, (**c**) TEM micrograph, (**d**) EDS and (**e**) SAED pattern.

The details of as-SPSed Cu-2Cr-1Nb alloy have been described in our previous work [41]. The microstructural features of as-SPSed alloy are briefly described in Figure 1. The second phase was Cr_2_Nb with size of 0.15–0.50 μm, the average of which was 0.33 μm (Figure 1a); only a small amount of Cr_2_Nb phase with size of below 0.15 μm was observed (Figure 1b). The TEM observation results showed that a small amount of the nano-Cr_2_Nb phase with a size of 30–100 nm, distributed in the matrix uniformly, besides submicron phase with a size of 0.1–0.3 μm; no Cr_2_Nb phase with a size of less than 30 nm was observed (Figure 1c). The energy dispersive spectrum (EDS) (Figure 1d) and selected area electron diffraction (SAED) (Figure 1e) results showed that the second phase with size of 30–100 nm (the square area in Figure 1c) was Cr_2_Nb phase with face centered cube (*FCC*) structure (Figure 1e).

### 3.2. The Microstructure of the Heat-Treated Cu-2Cr-1Nb Alloy

**Figure 2:**
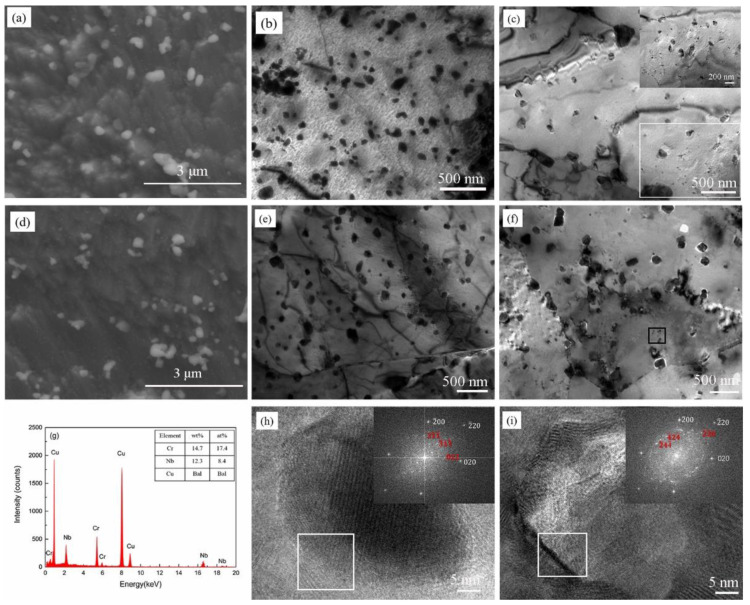
Microstructure of Cu-2Cr-1Nb alloy heat-treated at 500 °C for (**a**–**c**) 1 h and (**d**–**i**) 2 h.

**Figure 3:**
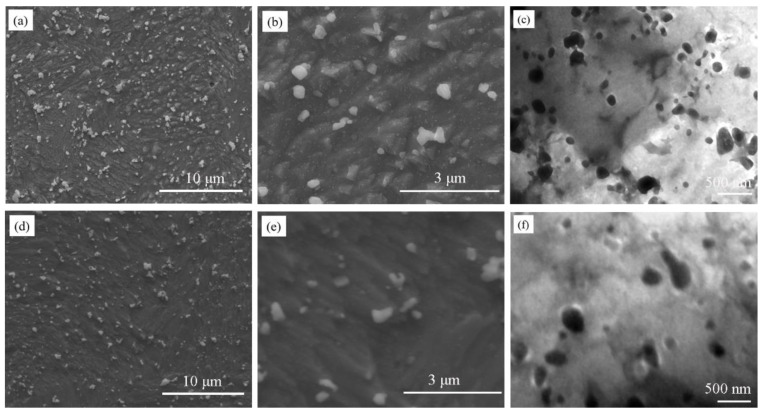
Microstructure of Cu-2Cr-1Nb alloy heat-treated for 1 h at different temperatures; (**a**–**c**) 700 °C and (**d**–**f**) 950 °C.

**Figure 4:**
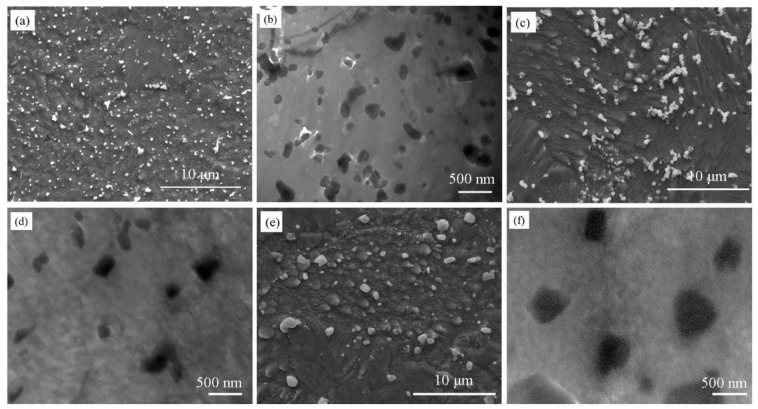
Microstructure of Cu-2Cr-1Nb alloy heat-treated for 72 h at different temperatures; (**a**,**b**) 500 °C, (**c**,**d**) 700 °C and (**e**,**f**) 950 °C.

Figure 2, Figure 3 and Figure 4 are the microstructures of the Cu-2Cr-1Nb alloy heat-treated at different temperatures for different times. The heat treatment temperature and time had important effects on microstructure of the alloy. Figure 2 shows the microstructure of the alloy heat-treated at 500 °C for 1 h and 2 h. After heat treatment for 1 h, Cr_2_Nb phase with sizes of 0.15–0.50 μm and less than 0.15 μm was observed in the alloy, its average size was below 0.33 μm, and the number of Cr_2_Nb phase with size of less than 0.15 μm was significantly more than that of SPSed alloy (Figure 2a). The TEM observation results showed that, after heat treatment at 500 °C for 1 h, Cr_2_Nb phase with size of less than 30 nm was also observed, besides Cr_2_Nb phase with sizes of 0.1–0.3 μm and 30–100 nm, and the number of fine Cr_2_Nb phase was more than that of SPSed alloy (Figure 2b,c). After heat treatment at 500 °C for 2 h, the size and number of Cr_2_Nb phase were not changed obviously (Figure 2d–f), compared with the alloy heat treated at 500 °C for 1 h (Figure 2a–c). The EDS result showed that the atomic ratio of Cr and Nb of the second phase (less than 30 nm, the square area in Figure 2f) in the alloy heat-treated at 500 °C for 2 h was about 2:1, which was similar to Cr_2_Nb (Figure 2g). Figure 2h,i are high-resolution transmission electron microscopy (HRTEM) images of the alloy heat-treated 500 °C for 2 h. The Fourier transform and SAED calibration results demonstrated that the second phase, with a size of less than 30 nm, had *FCC* structure. The above results indicate that multi-scale Cr_2_Nb phase with *FCC* structure was formed after brief heat treatment at 500 °C.

Figure 3 is the microstructure of Cu-2Cr-1Nb alloy heat-treated for 1 h at 700 °C and 950 °C, the fine Cr_2_Nb coarsened while the number decreased, and the temperature had an important influence on the size and number of Cr_2_Nb phase. After heat treatment at 700 °C for 1 h, Cr_2_Nb phase with sizes of 0.15–0.50 μm and less than 0.15 μm was also observed. However, the size of fine Cr_2_Nb phase (less than 0.15 μm) increased while the number decreased (Figure 3a,b), compared with that in the alloy heat-treated at 500 °C for 1 h and 2 h (Figure 2a,d). The TEM observation result showed that multi-scale Cr_2_Nb phase with size of 40–300 nm was also observed in the alloy heat-treated at 700 °C for 1 h (Figure 3c). Increasing heat treatment temperature to 950 °C, the Cr_2_Nb phase with a size of 0.15–0.50 μm did not grow significantly (Figure 3d,e). However, the fine Cr_2_Nb phase (less than 0.15 μm) was obviously larger than that in the alloy heat-treated 500 °C and 700 °C for 1 h (Figure 2a and Figure 3a,b). The TEM observation result showed that Cr_2_Nb phase with a size of less than 80 nm was not observed, and the number of fine Cr_2_Nb phase was significantly reduced (Figure 3f).

Figure 4 shows the microstructure of Cu-2Cr-1Nb alloy heat-treated at different temperatures for 72 h. Both submicron and nano-Cr_2_Nb phase coarsened; the size of Cr_2_Nb phase increased with the increase of temperature, while the number decreased. After heat treatment for 72 h at 500 °C, 700 °C and 950 °C, the average sizes of submicron Cr_2_Nb phase of Cu-2Cr-1Nb alloy increased to 0.35 μm, 0.48 μm and 0.60 μm, respectively (Figure 4a,c,e). The TEM observation results showed that a small amount of Cr_2_Nb phase with a size of less than 100 nm was observed in the alloy heat-treated at 500 °C for 72 h (Figure 4b), but not observed in the alloy heat-treated at 700 °C and 950 °C for 72 h (Figure 4d,f).

Due to the combination of rapid solidification of close-coupled argon gas atomization and rapid densification of SPS, the Cr and Nb of Cu-2Cr-1Nb alloy existed in two ways of solid solution atoms and Cr_2_Nb phase [40,41]. The solid solubility of Cr and Nb and diffusivity of Nb in the copper matrix were the key factors to determine the growth rate and size of Cr_2_Nb phase.

When the alloy was heat-treated at 500 °C, the solid solubility of Cr and Nb in copper matrix decreased, and the diffusivity of Nb in copper matrix was low (1.87 × 10^−17^ cm^2^·s^−1^) [44]. After heat treatment at 500 °C for 1 h and 2 h, the Cr and Nb atoms were precipitated from the copper matrix to form multi-scale Cr_2_Nb phase with sizes of 0.1–0.5 μm, 30–100 nm and less than 30 nm (Figure 2). Prolonging heat treatment time to 72 h, the Cr_2_Nb phase grew up while the number decreased, and the average size increased (from 0.33 μm to 0.35 μm). Raising heat treatment temperature to 700 °C, the diffusivity of Nb was 3100 times [44] higher than that at 500 °C, and the growth rate of Cr_2_Nb phase increased significantly. After heat treatment at 700 °C for 1 h, the fine Cr_2_Nb phase coarsened (Figure 3a–c). Changing heat treatment time to 72 h, both submicron and nano-Cr_2_Nb phases coarsened (Figure 4c,d). When heat treatment temperature was 950 °C, the solid solubility and diffusivity of Cr and Nb in copper matrix, and the growth rate of Cr_2_Nb phase increased obviously, Cr_2_Nb phase grew further (Figure 3 and Figure 4). However, even at 950 °C, the intermetallic compound Cr_2_Nb has good thermal stability, the diffusivity of Nb in copper matrix was less than 4.08 × 10^−11^ cm^2^·s^−1^ [44], and the growth rate of Cr_2_Nb phase was still low. Therefore, the average size of Cr_2_Nb phase of Cu-2Cr-1Nb alloy increased from 0.33 μm to 0.60 μm after heat treatment for 72 h at 950 °C (Figure 4e). These results indicate that the multi-scale Cr_2_Nb phase was formed by heat treatment at 500 °C. The Cr_2_Nb phase could be effectively regulated by the combination of rapid solidification of close-coupled argon gas atomization, rapid densification of SPS and heat treatment, and the Cr_2_Nb phase was significantly finer than that in Cu-8Cr-4Nb [13,37], Cu-4Cr-2Nb [13] and Cu-8Cr-4Nb (Zr) [31] alloys.

### 3.3. The Properties of Cu-2Cr-1Nb Alloy

**Figure 5:**
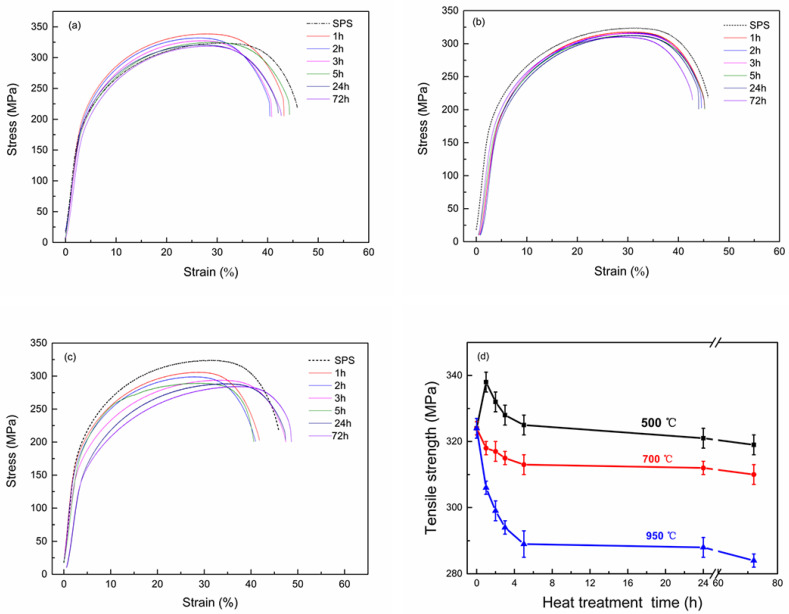
Stress–strain curves of Cu-2Cr-1Nb alloy heat-treated for different times at (**a**) 500 °C, (**b**) 700 °C and (**c**) 950 °C; (**d**) the corresponding tensile strength.

Figure 5 shows the stress–strain curves and room temperature tensile strength of the Cu-2Cr-1Nb alloy. The stress-strain curves of heat-treated alloy in this work were similar to that of SPSed alloy, while the tensile strengths were different. The heat treatment hardening occurred at the beginning of heat treatment at 500 °C, and the peak value of tensile strength (338 MPa) was appeared after for 1 h, which was 4.3% higher than that of the SPSed alloy. Then, the tensile strength of the alloy decreased with the increase of heat treatment time. After heat treatment for 2 h and 5 h, the tensile strength of the alloy was 332 MPa and 325 MPa, respectively. However, it was no longer significantly reduced with the increase of heat treatment time. After heat treatment for 72 h at 500 °C, the tensile strength of the alloy was 319 MPa, 1.5% lower than that of the SPSed alloy. Changing heat treatment temperature to 700 °C and 950 °C, the heat treatment softening phenomenon was appeared, and the tensile strength of the heat-treated alloy decreased with the increase of heat treatment temperature and time. After heat treatment for 1 h, the tensile strength of the alloy decreased from 324 MPa to 318 MPa and 306 MPa, 1.9% and 5.6% lower than that of the SPSed alloy. Increasing heat treatment time to 5 h, the tensile strength was 313 MPa and 289 MPa, which were 3.4% and 10.8% lower than that of the SPSed alloy; and the tensile strength of the alloy heat-treated for 72 h decreased by 4.3% and 12.3%, respectively, compared with that of the SPSed alloy.

The strength of the alloy was closely related to the microstructure, especially content, size, distribution of Cr_2_Nb phase and the solid solution Cr and Nb atoms. The contributions to the strength of Cu-2Cr-1Nb alloy were fine grain strengthening, solution strengthening and precipitation strengthening. The Cr_2_Nb phase formed during close-coupled argon atomization and SPS can effectively inhibit the growth of matrix grain, and the fine grain strengthening effect did not change significantly after heat treatment [13,26,36,37]. The main contributions to the change of alloys’ strength were solid solution strengthening and precipitation strengthening. The solid solution strengthening was mainly related to the solid solubility of Cr and Nb atoms. The precipitation strengthening was mainly determined by the amount, size and distribution of Cr_2_Nb phase [13,15,25,45]. 

After heat treatment at 500 °C for 1 h and 2 h, Cr and Nb precipitated from the solid solution to form multi-scale Cr_2_Nb phase, and the number of Cr_2_Nb phase increased (Figure 2), and the precipitation strengthening effect was enhanced by the synergistic effect of nano and submicron Cr_2_Nb phase, while the solid solution strengthening effect of Cr and Nb atoms decreased. However, the contribution of precipitation strengthening effect to the alloy’s strength was greater than that of the solid solution strengthening; therefore, the tensile strength of the alloy increased, and the alloy showed a heat treatment hardening phenomenon. 

As shown in Table 2, after heat treatment at 500 °C for 2 h, the tensile strength of the alloy at 700 °C was 76 MPa, which was higher than that of Cu-8Cr-4Nb alloy prepared by gas atomization, VHP and aging [33,37], equivalent to Cu-4Cr-2Nb alloy [13]. With the increase of heat treatment time, the precipitated nano-Cr_2_Nb phase coarsened, while the precipitation strengthening effect of Cr_2_Nb phase reduced, and the alloy’s strength decreased. After heat treatment at 500 °C for 72 h, the number of fine Cr_2_Nb phase decreased, while the average size of tCr_2_Nb phase increased from 0.33 μm to 0.35 μm (Figure 4a,b), and the strength of the alloy decreased by 1.5% compared with the as-SPSed alloy. 

After heat treatment at 700 °C for 1 h, the Cr_2_Nb phase coarsened (Figure 3a–c), and the precipitation strengthening effect of Cr_2_Nb phase decreased; the strength of the alloy was reduced and lower than that of the SPSed alloy (from 324 MPa to 318 MPa). Prolonging heat treatment time to 72 h, Cr_2_Nb phase grew, and the average size of Cr_2_Nb phase increased to 0.48 μm, some of Cr_2_Nb phase agglomerated, and the spacing between Cr_2_Nb phases increased (Figure 4c,d). Therefore, the precipitation strengthening effect of Cr_2_Nb phase and the strength of the alloy decreased. 

Increasing heat treatment temperature to 950 °C (same as the SPS), the fine Cr_2_Nb phase, which was formed during close-coupled argon gas atomization and SPS, was shrunk and disappeared, while the large Cr_2_Nb phase coarsened; the number and precipitation strengthening effect of Cr_2_Nb phase decreased, thus the strength of the alloy was lower than that of the SPSed alloy. After heat treatment at 950 °C for 1 h, the tensile strength of the alloy reduced by 10.8%. However, even after heat treatment at 950 °C for 72 h, the coarsening of the large Cr_2_Nb phase was not significant (from 0.33 μm to 0.60 μm, Figure 4e), and the reduction of the strength was not evident; the strength retention rate was 87.7%. In a word, after brief heat treatment at a low temperature, the alloy showed heat treatment hardening; increasing temperature and time of heat treatment, it exhibited heat treatment softening.

**Figure 6:**
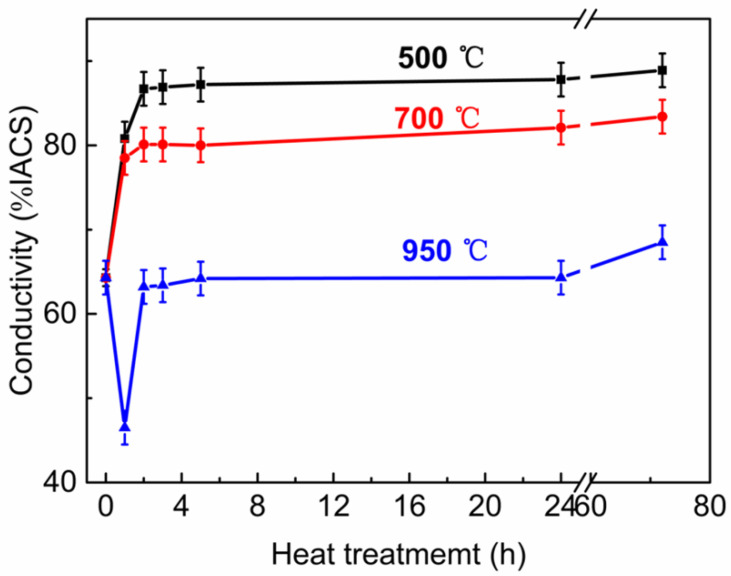
Conductivity of Cu-2Cr-1Nb alloy heat-treated at different temperatures for different times.

Figure 6 shows the conductivity of the Cu-2Cr-1Nb alloy. When the alloy was heat-treated at 500 °C and 700 °C, the conductivity of the alloy increased rapidly with the increase of heat treatment time at the early stage. After heat treatment for 1 h, the conductivity of the alloy increased from 64.3% IACS to 80.8% IACS and 78.5% IACS, respectively, which were 25.7% and 22.1% higher than that of the SPSed alloy. When heat treatment time was further increased, the conductivity of the alloy increased slowly. The conductivity of the alloy heat-treated for 2 h was 86.7% IACS and 80.1% IACS, respectively. Increasing heat treatment time to 5 h, the conductivity of the alloy was 87.2% IACS and 80% IACS, respectively, which were 35.6% and 24.4% higher than that of the SPSed alloy. When heat treatment time was more than 5 h, the conductivity was no longer significantly increased. The conductivity of the alloy heat-treated for 72 h was 88.9% IACS and 83.4% IACS, 38.3% and 29.7% higher than that of the SPSed alloy. 

When heat treatment temperature was 950 °C, the conductivity of the alloy decreased with the increase of heat treatment time at the early stage. The conductivity of the alloy heat-treated for 1 h was 46.5% IACS, 27.7% lower than that of the SPSed alloy. The conductivity then raised with the increase of time. The conductivity of the alloy heat-treated for 2 h was 63.2% IACS, which was 1.7% lower than that of the SPSed alloy. With the increase of heat treatment time, the conductivity of the alloy did not change obviously, and the conductivity of the alloy heat-treated for 72 h was 68.5% IACS, which was 6.5% higher than that of the SPSed alloy.

The conductivity of the copper alloy was mainly affected by the impurity, interface, dislocation, phonon and so on [13]. In this work, the conductivity of Cu-2Cr-1Nb alloy was tested at room temperature. The relative density of the prepared Cu-2Cr-1Nb alloy was as high as 99.8% [40]. The effect of porosity, interface between powders, phonon and dislocation on conductivity of the alloy was small. The microstructure (including solid-solution Cr, Nb atoms and Cr_2_Nb phase) was the key factor affecting the conductivity of the alloy. The sintering temperature of SPS was as high as 950 °C, the solid solubility of Cr and Nb was relatively high, so the conductivity was low (64.3% IACS). 

After heat treatment at 500 °C, some Cr and Nb atoms precipitated from copper matrix to form Cr_2_Nb phase, and the solid solubility of Cr and Nb was reduced. The content of solid-solution atoms and their effect on electron scattering decreased, so the conductivity of the alloy increased. After heat treatment at 500 °C for 2 h, the conductivity of the alloy reached 86.7% IACS, which was 34.8% higher than that of the SPSed alloy, 60.6%, 15.6% and 17.2% higher than that of HE/aged Cu-8Cr-4Nb alloy [13], VHP/aged Cu-8Cr-4Nb and Cu-4Cr-2Nb alloy [37], respectively, as indicated by Table 2. The strength and conductivity of Cu-2Cr-1Nb alloy had been improved simultaneously, and the good match of strength and conductivity of Cu-Cr-Nb alloy was obtained. Increasing heat treatment time, the solid-solution Cr and Nb atoms further precipitated to form Cr_2_Nb phase, and the Cr_2_Nb phase coarsened. The number of Cr_2_Nb phase, interface between Cr_2_Nb phase and the matrix, and their effect on electron scattering decreased, so the alloy’s conductivity increased. After heat treatment at 700 °C, the solid solubility of Cr and Nb in copper matrix was higher than that of the alloy heat-treated at 500 °C, and the conductivity was lower. 

When heat treatment temperature was 950 °C, the fine Cr_2_Nb phase formed during SPS was re-dissolved into the copper matrix at the early stage of heat treatment, the solid solubility of Cr and Nb atoms increased, and the conductivity of the alloy decreased (46.5% IACS). With the increase of heat treatment time, the micropore in the alloy further shrunk and disappeared, and its effect on electron scattering was weakened [46,47], and the number of Cr_2_Nb phase and the interface between Cr_2_Nb phase and the matrix reduced due to Cr_2_Nb phase coarsened, while the conductivity of the alloy increased. However, the solid solubility of Cr and Nb atoms at 950 °C is higher than at 500 °C and 700 °C, the conductivity of the alloy after heat treatment at 950 °C was lower than that at 500 °C and 700 °C.

## 4. Conclusions

In the present work, the effects of heat treatment on the Cr_2_Nb phase and the properties of the SPSed Cu-2Cr-1Nb (at%) alloy were investigated. The main findings are summarized as follows:

(1) The Cr_2_Nb phase of the Cu-2Cr-1Nb alloy could be regulated by heat treatment. The multi-scale Cr_2_Nb phase with sizes of 0.10–0.50 μm, 30–100 nm and less than 30 nm was formed after heat treatment at 500 °C for 1 h and 2 h; a small amount of Cr_2_Nb phase with a size of less than 100 nm was observed in the alloy heat-treated at 500 °C for 72 h. With the increase of heat treatment temperature and time, the Cr_2_Nb phase coarsened; the average size of Cr_2_Nb phase of the alloy heat-treated for 72 h at 700 °C and 950 °C increased from 0.33 μm to 0.48 μm and 0.60 μm, and Cr_2_Nb phase with a size of less than 100 nm was not observed.
(2) The strength and conductivity of the Cu-2Cr-1Nb alloy were simultaneous improved by heat treatment, and they were 332 MPa and 86.7% IACS, respectively, which were 2.5% and 34.8% higher than that of SPSed alloy, and the tensile strength at high temperature 700 °C was 76 MPa, after heat treatment at 500 °C for 2 h. Increasing heat treatment temperature and time, the tensile strength of the alloy was reduced by 1.5%, 4.3% and 12.3% after heat treatment at 500 °C, 700 °C and 950 °C for 72 h.
(3) These suggest that the simultaneous improvement of strength and conductivity of Cu-Cr-Nb alloy can be realized by reducing content of alloying elements Cr and Nb, rapid solidification by close-coupled argon gas atomization, and rapid densification by SPS and heat treatment. The good matching of strength, conductivity and thermal stability of Cu-Cr-Nb alloy can also be achieved.

## Figures and Tables

**Table 1:** Chemical composition of close-coupled argon gas atomized Cu-2Cr-1Nb alloy powder.

Element	Cr	Nb	O	Cu
Measured composition (wt%/at%)	1.54/1.89	1.26/0.88	0.01/0.02	Bal

**Table 2:** Comparison of properties of Cu-Cr-Nb alloy.

Alloy	Processing Method	Phase Size (μm)	Tensile Strength (MPa)		Conductivity (% IACS)	Refs.
			25 °C	700 °C		
Cu-0.47Cr-0.16Nb (wt%)	Casting + Homogenization + CR + Aging	0.70	453	-	89.1	[15]
Cu-2Cr-1.35Nb-0.15Zr (wt%)	Drop casting + Deformation processing + Heat treatment	0.3–1	385	-	<60	[2]
Cu-8Cr-4Nb (at%)	Casting	0.7–7	-	-	-	[29,30]
	Atomization + HE + Aging	0.93	426	100	54	[13,22]
	Atomization + VHP + Aging		400	72	75	[33,37]
Cu-4Cr-2Nb (at%)	Atomization + HE + Aging	0.78	325	75	74	[13]
Cu-2Cr-1Nb (at%)	Close-coupled gas atomization + SPS + Aging	<0.33	332	76	86.7	This work
